# Clinical implication of low estrogen receptor (ER-low) expression in breast cancer

**DOI:** 10.3389/fendo.2022.1015388

**Published:** 2022-11-24

**Authors:** Tomás Reinert, Fanny Cascelli, Cristiano Augusto Andrade de Resende, Aline Coelho Gonçalves, Vania Sanchez Prette Godo, Carlos Henrique Barrios

**Affiliations:** ^1^ Breast Medical Oncology, Oncoclínicas, Porto Alegre, Brazil; ^2^ Breast Cancer Group, Latin American Cooperative Oncology Group, Porto Alegre, Brazil; ^3^ Breast Medical Oncology, Oncoclínicas, São Paulo, Brazil; ^4^ Breast Medical Oncology, Oncoclínicas, Brasília, Brazil; ^5^ Breast Medical Oncology, Oncoclinicas, Rio de Janeiro, Brazil

**Keywords:** breast cancer, estrogen receptor, endocrine therapy, ESR1, ER low positive

## Abstract

Breast cancer is a heterogeneous disease, and the estrogen receptor (ER) remains the most important biomarker in breast oncology. Most guidelines set a positive expression threshold of 1% staining in immunohistochemistry (IHC) to define ER positivity. However, different expression levels may be associated with diverse degrees of sensitivity to endocrine therapy as ER expression may impact breast cancer molecular biology as a continuous variable. ER^-lo^ tumors, defined as those with 1-10% ER expression, represent a relatively small subgroup of breast cancer patients, with an estimated prevalence of 2-7%. These tumors are similar to ER^neg^ disease in their molecular landscape, clinicopathological characteristics, prognosis, and response to therapy. Nevertheless, a proportion may retain some degree of ER signaling dependency, and the possibility of responding to some degree to endocrine therapy cannot be completely ruled out. This review article discusses the most important considerations regarding the definition of ER positivity, pathology assessment, prognosis, and therapeutic implication of ER^lo^ breast cancer from the medical oncology perspective.

## Introduction

More than 60 years after its discovery, the estrogen receptor (ER) remains the most important biomarker in breast oncology. ER status is essential in clinical decisions and predicting outcomes for breast cancer (BC) patients. ER-targeted therapies have significantly improved the clinical outcomes in patients with ER-positive (ERpos) BC. The expression of estrogen receptors indicates a biological dependency on estrogen signaling, and notably, therapeutic effects are correlated with ER levels in tumor cells (1).

Most guidelines set a positive expression threshold of 1% staining to define ER positivity. However, different expression levels may result in different responses and outcomes as ER expression may impact breast cancer molecular biology as a continuous variable. Tumors with lower ER expression may be biologically distinct from high ER expressing cases, with different behavior and degree of sensitivity to systemic therapies, especially endocrine therapy (ET) and chemotherapy (CT).

Aligned with this, the latest ASCO/CAP guideline recommends reporting tumors with 1 to 10% ER expression as a distinct subgroup, entitled “ER Low Positive” (ERlo), recognizing the limited benefit of ET in this subset of tumors. Available evidence indicates that these patients have clinical outcomes and molecular biology comparable to ER-negative (ERneg) cancers (2). In this brief review, we will discuss important issues regarding the definition of ER positivity, describing the clinical and molecular characteristics of ERlo BC, with a particular focus on therapeutic implications from the clinical oncologist’s point of view.

## Definition of ER-positivity

In 1999, Allred and colleagues reported that the ER status by immunohistochemistry (IHC) is superior to the ligand-binding assay (LBA) for predicting outcomes with ET in BC (3). In the Allred scoring system, a proportion score was initially assigned, which represented the estimated proportion of positive-staining tumor cells (0, none; 1, < 1%; 2, 1-10% 3, 10-33%, 4, 33-66%; 5, >66%). Next, an intensity score was assigned, which represented the average intensity of positive tumor cells (0, none; I, weak, 2, intermediate; and 3, strong). The proportion and intensity scores were then added to obtain a total score, which ranged from 0 to 8. An IHC score of greater than 2 (corresponding to as few as 1-10% weakly positive cells) was used to define ER positivity based on a univariate cut-point analysis of all possible scores and disease-free survival (DFS) in patients receiving adjuvant ET. Using this definition, 71% of tumors were defined as ERpos by IHC. The level of agreement with LBA was 86%. Notably, this publication proposed the cut-off of IHC score >2 for predicting improved outcomes, and this value was used in subsequent studies. In this pivotal study, 16% of patients with a lower level (IHC Score 3-4) and 40.8% with an intermediate level (IHC Score 5-6) had inferior survival outcomes compared to the 25.6% of patients with a high-level ER (IHC Score 7-8) (3).

Importantly, and probably related to an increased sensitivity of current IHC assays, the proportion of patients classified as having low, intermediate, or high levels of ER expression has changed over time. Nowadays, the majority of ERpos breast tumors have high expression of ER (ERhi). Unlike the initial publications reported, the current prevalence of ERpos breast cancer with weak and intermediate is less than 20% (4). We can hypothesize that many tumors classified as low or intermediate in older studies, with less sensitive assays, would now be considered ERhi. Consequently, benefits observed in the ERlo population in older trials might have been related to misclassification.

The 2010 ASCO/CAP guideline considered a tumor as ERpos if as little as 1% of tumor nuclei stained positively by IHC (5). This was supported by data that patients with a low level of receptor positivity (1-10%) still could benefit from ET and have better outcomes than those with ERneg tumors (6). However, the cut-off value for ER positivity has long been controversial since other studies support a higher cut-off point, and in clinical practice, a range of thresholds are used to establish hormone receptor positivity (6). The controversy is well aligned with the fact that ER-positive tumors are heterogeneous, have different degrees of endocrine sensitivity, and variable responses to ET, and at least a subset has molecular characteristics and outcomes comparable to tumors without ER expression. To that end, in cases with 1-10% positive tumor cell nuclei, the 2020 ASCO/CAP Guidelines recommends a new category: ER-low-positive (ERlo) BC. Notably, the guideline also comments on the limited available data on the benefit of ET in these patients.

While most breast cancers show either strongly ER-positive staining or a complete absence of ER staining, a few cases are found with low receptor expression levels (6) (**Table 1**). The expression pattern probably reflects each tumor’s higher versus lower biological dependency on estrogen signaling pathways. As shown in **Table 1**, according to a variety of published studies evaluating more than 100.000 breast cancer patients, only about 2-7% of breast tumors fit the ERlo category.

**Table 1:** Prevalence of ER^lo^ breast cancer in selected publications published within the last ten years.

	Study design	N	ER^lo^ (%)	ER^hi^ (%)	ER^neg^(%)
Yoon 2021 (7)	Retrospective	2.162	2.5%	76.5%	21%
Villegas 2021 (8)	Retrospective	2.765	3%	64%	33%
Fujii 2017 (9)	Retrospective	3.055	5.6%	63.9%	30.5%
Fei 2021 (1)	Retrospective	4.179	2.3%	71.4%	26.3%
Luo 2022 (10)	Retrospective	5.466	5.1%	62.2%	32.7%
Paakkola 2021 (11)	Meta-analysis	7.431	7%	60%	33%
Yi M 2014 (12)	Retrospective	9.639	2.6%	80.5%	16.9%
Chen 2018 (13)	Meta-analysis	16.606	5%	69.9%	25.1%
Cai 2022 (14)	Retrospective	22.768	4.4%	NR	NR
Schrodi 2021 (15)	Retrospective	38.560	2%	85%	13%

It is important to emphasize that the current classification and recommendations of international guidelines for the definition of ER positivity are based on traditional histopathology and immunohistochemistry assessment, according to the ASCO/CAP criteria (16). Even though there are multiple studies that compare ER gene expression (messenger RNA) with IHC with relatively good agreement, the ASCO/CAP guideline agreed that this was insufficient to recommend these assays in routine clinical 1practice (17, 18). As discussed afterwards, advances in digital pathology integrated with the use of artificial intelligence algorithms are likely to revolutionize this classification in the near future (19).

## Pathology assessment and diagnostic workflow

**Figure 1:**
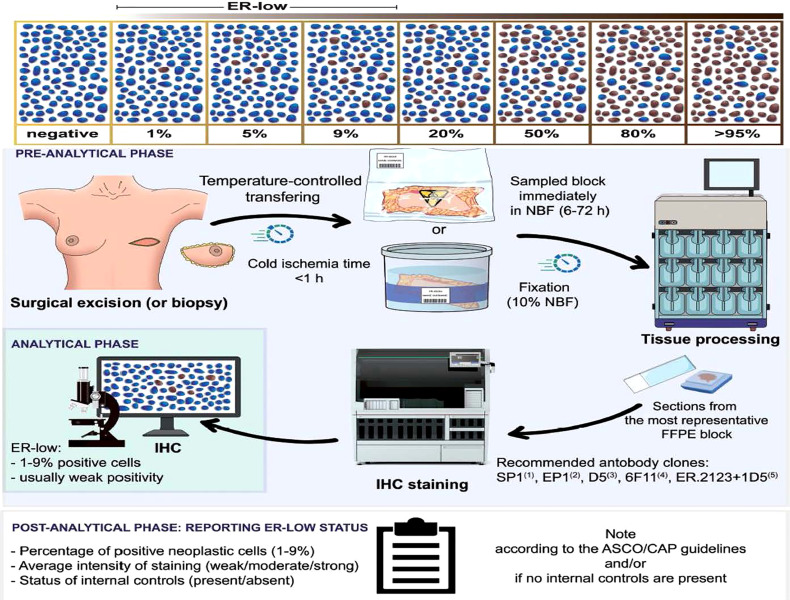
Reprinted with permission from Fusco N et al., 2021 (16). Schematic representation of the standard operating procedures for an appropriate low ER status assessment. After the bioptic or surgical excision, the sample should be transferred to the pathology lab using a temperature-controlled system. Of note, the cold ischemia time should not exceed 1 hour. The preservation of the sample for transport can be either under vacuum or in 4% neutral buffered formalin. The time before sampling should range from 6 to 72 hours. After tissue processing, the pathologist should select the most representative sample and be subjected to immunohistochemistry for analysis, which can rely on validated digital pathology tools. The biomarker report in case of low ER positivity requires information on the percentage of positive neoplastic cells, staining intensity, and status of the internal controls. According to the ASCO/CAP guidelines, a note should be added for all ER^lo^ tumors. ER, estrogen receptor; NBF, neutral buffered formalin; FFPE, formalin-fixed paraffin-embedded; ASCO, American Society of Clinical Oncology; CAP, College of American Pathologists.

Optimizing the diagnostic workflow, with strict procedures in both the preanalytical and analytical phases, quality control, focused training programs, adequate post-analytical processes, and harmonization studies, is essential to minimize the number of false-positive and false-negative for any IHC biomarker, and the same applies to ER expression (see **Figure 1**). These issues were initially identified during the enrollment of hormone therapy trials, after central laboratory testing, with a frequency ranging from 10 to 20%.

Several technical issues have been proposed as influencing ER expression determination. Most ER falsely-negative cases were related to preanalytical problems, such as poor fixation (20). This led to an initial discussion questioning if ERlo breast cancer was an actual biological entity or just an artifact of surgical pathology (21). At the same time, the dynamic range of ER expression in normal epithelium around ERlo BC cells was significantly lower than in other ER-positive breast tumors, suggesting weakness of the staining process rather than a decrease in biological ER expression in those tumors (4). Loss of tissue quality was also associated with loss of ER expression measured by quantitative immunofluorescence (22). Other studies suggest that loss of ER expression could be caused by an extended cold ischemic time (23).

At the same time, intratumoral heterogeneity is a well-known feature of breast cancer that is associated with therapeutic and diagnostic implications, especially in the context of immunohistochemical evaluation of hormone receptors, Ki67 and HER2 (15).

Nevertheless, these facts do not explain the similarity between ERlo tumors and TNBC, and cumulative evidence strongly suggests that ERlo breast cancer is an existing entity.

Few studies have addressed the interobserver agreement of ERlo expression status. In a pilot multi-platform study on 264 breast cancers, a 5% discrepancy rate was reported for ER assessment, with most discordant cases falling in the ERlo range. Additionally, some cases showed weak staining, probably due to the low sensitivity of the IHC assay’s clone (24).

Important considerations regarding precise methodologies for pathological identification of ER status are summarized in **Figure 1**. These issues have been comprehensively reviewed elsewhere (16, 20, 25) and are beyond the scope of this manuscript.

## Clinicopathological features and molecular characterization of ER^lo^

Various studies have provided a good panorama on the anatomopathological characteristics, biological behavior, and molecular profile of ERlo breast cancer; some of this data is summarized in **Table 2**. Large studies of population-based cohort analysis (8, 26), suggest that ER low-expression is significantly associated with a higher proportion of younger patients with grade 3 tumors. Also, show a lower prevalence of smaller primaries (<2 cm) and lobular histology compared with ERhi breast cancers. An increased presence of tumor-infiltrating lymphocytes (TILs) and a higher Ki67 index have also been described (20). Interestingly, no statistical difference in clinicopathological characteristics was observed when ERlo tumors were compared with ERneg cases.

**Table 2:** Summary of clinical, pathological and molecular characteristics of ER^lo^ breast cancer and comparison of these characteristics in ER^hi^ and ER^neg^ tumors.

		TNBC	ER^LO^(ER+1-10%)	ER^hi^(ER+>10%)
**MUNICH CANCER REGISTER**				
N		3,364	553	29,449
Age <50		33%	36%	20%
c/pT1		39%	38%	59%
Grade 3		75%	71%	16%
Invasive Lobular Carcinoma		2%	5%	16%
**FLATIRON HEALTH RECORD**				
N		634	83	3,980
Premenopausal		20%	25%	17%
c/pT1		17%	17%	19%
Grade 3		80%	74%	17%
Invasive Lobular Carcinoma		1.6%	3.6%	14%
**MD ANDERSON CANCER CENTER COHORT**				
N		183	31	251
Molecular Subtypes by PAM50	Luminal A	2 (1%)	2 (6%)	120 (48%)
	Luminal B	1 (0.5%)	3 (10%)	61 (24%)
	HER2 Amplified	51 (28%)	9 (29%)	38 (15%)
	Basal	111 (60.5%)	13 (42%)	16 (6.5%)

The clinicopathological complexity of ERlo tumors reflects their molecular heterogeneity. Accumulating data suggested a series of molecular mechanisms of ER expression loss during cancer evolution that can result in weak and limited ER expression. These mechanisms include ESR1 gene mutation or loss of heterozygosity, epigenetic modulation, post-transcriptional regulation, and upregulation of growth factor signaling inducing hyperactive mitogen-activated protein kinase (MAPK), hence leading to a reversible loss of ER expression (21).

Regarding the genetic profile, in an evaluation of 314 patients from the MD Anderson Cancer Center (MDACC), it was observed that the incidence of germline BRCA mutations is similar in patients with ERlo and TNBC (triple-negative breast cancers (39.5 vs. 36.1%, respectively), with a predominance of BRCA1 in both tumor profiles (27). This is an essential issue since these patients may not undergo adequate genetic counseling without additional risk factors, and a correct diagnosis of hereditary breast cancer syndromes can be missed.

The same group published a series with 465 patients with stage I - III breast cancer, demonstrating that the presence of estrogen receptor gene (ESR1) mRNA was documented in only 24% of tumors that express ER from 1 to 9%, while in tumors with ER above 10% the rate of ESR1 mRNA positivity was 92% (28). Molecular evaluation of the intrinsic subtype by PAM50 of these patients was also performed, showing the presence of a basal subtype in 60.5%, 42%, and 6.5% of the ER-negative, ERlo,and ERhi tumors, respectively. Villegas et al. (29) also analyzed the transcriptomic profile of 2,765 patients with HER2-negative early breast cancer who were randomized in the GeparQuinto and GeparSepto neoadjuvant clinical trials. RNA sequencing was performed in 38 tumors with low estrogen receptor expression, and a basal-like molecular subtype was identified in this population in 87% of cases.

A recent study evaluated the Oncotype DX (ODX) recurrence score genomic assay in 38 patients with ERlo HER2-negative early breast cancer: approximately 95% of cases had a recurrence score (RS) above 25, and no case presented RS below 21 (11). These figures are clearly different from those seen in tumors with higher ER expression, where most cases have low or intermediate RS (9, 10). Therefore, ODX and other similar gene expression tests are probably of limited utility if ERlo BC, as these data indicate that most would benefit from (neo)adjuvant chemotherapy.

Yoder et al., recently presented data on a cohort of 516 patients with stage I-III HER2-negative breast cancer and ER/PR expression <10% (including ERlo and ERneg cases) who were enrolled in a multicenter prospective registry. Demographic, clinical, and treatment characteristics, including racial and ethnic distribution, the prevalence of gBRCAm, and chemotherapy use were not different between the TNBC and ERlo groups. No difference was observed in recurrence-free survival (RFS) and overall survival (OS). Among 358 patients that received neoadjuvant chemotherapy, rates of pathologic complete response (pCR) were similar for TNBC and ERlo groups (49% vs. 51%, respectively, p=0.8) (30)

These data together reinforce the concept that ERlo breast cancer is constituted by more proliferative tumors with high risk and worse prognosis, resembling TNBC.

## Prognosis

Various publications evaluated if ERlo status impacts prognosis and compared outcomes of this subgroup of patients with cohorts of ERneg and ERhi. Most published data propose that ERlo tumors have a worse prognosis than ERhi and comparable outcomes to ER-negative tumors.

Villegas et al. (29) observed no statistically significant difference concerning disease-free survival (DFS) and OS between patients with ERlo and ER neg tumors. Additionally, the study showed differences in risk between patients with ERlo tumors and those with ERhi tumors remained significant in multivariate analysis, indicating a higher probability of relapse, distant relapse, and death in women with ERlo breast cancer.

Furthermore, Pakkola et al. (31) confirmed these results in a meta-analysis that ERlo breast cancer was associated with worse DFS than ERhi breast cancer, and there was no statistically significant difference between ERlo and ERneg breast cancer in terms of DFS and OS. It is important to emphasize that even though this trend was demonstrated in other studies (32, 33), there are some conflicting reports. For example, in a cohort of 4,179 patients, Fei et al. showed that ERlo tumors had a favorable prognosis compared to TNBC and no difference in outcomes compared to the ERhi group (1).

The annual recurrence pattern of ERlo cases was similar to TNBC. Dowsett et al. reported that the recurrence rate of patients with ERlo tumors was higher in the first 5 years (1%) and reduced to 1-3% during 5-10 years after diagnosis (34). In contrast, the recurrence rates of patients with ERhi were relatively low in the first five years, but in years 5-10, they almost doubled (2.5-4%) and became higher than those with ERlo disease. This pattern is similar to that observed in TNBC disease patients.

The issue of ERlo tumors having a worse prognosis in comparison to ERhi gained more importance since the recent incorporation of ER status in the American Joint Committee on Cancer (AJCC) Pathological Prognosis Staging (PPS) together with progesterone receptor (PR), HER2, and grade. PPS shows a superior prognostication power than the traditional TNM anatomical staging. In the PPS, compared to the corresponding anatomical staging, it will be downstaged if a tumor expresses ER and/or HER2. However, there is a caveat as in the AJCC guideline, ER positivity was defined as expression in 1% or more of tumor cells, without segregation into ERlo and ERhi. As previously discussed, ERlo cases are biologically more similar to ERneg and show worse outcomes than ERhi. Thus, using the current approach in AJCC staging, there is a real risk of downstaging ERlo cases that behave more like ERneg cases biologically, potentially resulting in undertreatment (2).

## Therapeutic implications and recommendations

Several questions remain about the role of ERlo status in therapeutic decisions regarding systemic therapies in BC, both in the early-stage tumors and in metastatic disease.

In the neoadjuvant setting, especially in patients with locally advanced disease, the decision between upfront surgery, neoadjuvant chemotherapy (NACT), and neoadjuvant endocrine therapy (NET) is a frequent challenge in routine patient care, especially considering the lack of clinical trials directly comparing these two strategies (35).

In the adjuvant setting, even though the potential benefit of chemotherapy is more significant than in patients with ERhi tumors, there are limited data on the benefits of ET, especially with the incorporation of escalation strategies in adjuvant ET, such as the use of ovarian function suppression (OFS) in premenopausal patients and the use of the CDK4/6 inhibitor abemaciclib in high-risk patients.

At the same time, it is unclear how ERlo status should dictate changes in the therapeutic algorithm of metastatic BC. Should these cases be considered as ER-positive and treated preferentially with endocrine agents, or should we assume that these patients are mostly endocrine resistant and more chemo-sensitive? In parallel, the introduction of immunotherapy for TNBC raises the issue of the incorporation or not of these patients as this specific subgroup with low expression was not included in the pivotal trials.

In early-stage and locally-advanced breast cancer (LABC), Dieci et al. (13) evaluated clinical outcomes in patients with ERlo, HER2-negative, stage I-III breast cancer as compared to patients with TNBC undergoing NACT. In this study, primary breast cancer with ERlo status had similar clinical behavior to those with ER <1% (TNBC). Among the 165 patients who received NACT, the pathological complete response (pCR) rate was similar in ER-negative (38%) and ERlo patients (44%). There was no significant difference between RFS and OS in univariate and multivariate analyses. The 5-year iRFS was 74.0% in patients with ER-negative versus 73.1% in those with ERlo tumors (p = 0.6), while the 5-year OS was 82.3% and 76.7%, respectively (p = 0.8).

In patients with residual disease following NACT, the use of adjuvant capecitabine is the standard of care based on the data of the CREATE-X trial. It is important to emphasize that the study was positive in the intention to treat population and that, even though the benefit was more pronounced in the TNBC cohort [5y DFS TNBC cohort: HR=0.58 (IC 95% 0.39 - 0.87)], the majority of included patients had ER-positive tumors [5y DFS ER+ cohort: HR=0.84 (IC 95% 0.57 - 1.23)]. It is noteworthy that this strategy has been fully incorporated into clinical practice for patients with TNBC, but the use of capecitabine is not adopted for patients with ER-positive tumors. Although there have been no specific analyses for ER expression levels in this trial, we recommend that in patients with ERlo tumors and residual disease post-NACT, the use of adjuvant capecitabine should be strongly considered.

On the other hand, using NET should not be considered a preferential option in ERlo tumors. In a preclinical study, tamoxifen reduced epithelial cell volume in ERhi tumors but not in ERlo breast cancer (36). In the neoadjuvant P024, in which letrozole was compared with tamoxifen in the neoadjuvant setting, a linear association was observed between ET response rates and ER expression levels (37). Similar correlations between receptor expression and outcomes have been reported in other trials, such as the PADA-1 (38). Marginally ERpos tumors, defined as having an Allred score of 3-5, were still responsive to letrozole but not to tamoxifen. However, since the study only enrolled patients with ER>10% tumors, whether these findings can be extrapolated to ERlo tumors is unknown. A recently published evidence-based guideline for managing patients with primary ER-positive HER2-neg breast cancer clearly supported the use of ER and PR expression levels at diagnosis for triaging patients into three groups for the expected benefit of NET. The guideline stated that NET is likely to be inappropriate for the group of patients with tumors having an ER expression less than 40% (Allred <6) (39).

Endocrine therapy significantly benefits patients with ER-positive breast cancer but is not devoid of side effects. The collective lack of substantive evidence of benefit in ERlo tumors, associated with the potential adverse impact of ET on quality of life, contributes to low rates of adjuvant ET use in several cohorts (30). A lack of benefit of ET in patients with ERlo breast cancer was demonstrated in a meta-analysis, which enrolled six studies with more than 16,000 patients (40). Patients with ERlo breast cancer who received ET seemed to have a similar prognosis to those without any ET or those with ERneg cancer who received ET. In contrast, patients with ERhi tumors had better outcomes with ET compared with their ER 1-9% counterparts.

Also challenging are decisions regarding the indication of ovarian function suppression (OFS) as part of the adjuvant hormone therapy of premenopausal patients since the phase III SOFT, TEXT, and ASTTRA trials (41–43) excluded all patients with ER expression <10% and the vast majority of the patients included had tumors with an ER expression higher than 50%. Therefore, we do not have a representation of the ERlo population in studies addressing the role of OFS in premenopausal women.

Similar questions arise regarding the use of adjuvant CDK4/6 inhibitors in ERlo breast cancer. In the phase III monarchE trial, which included patients with ER-positive, HER2- negative, high-risk early breast cancer, abemaciclib combined with endocrine therapy significantly improved invasive DFS. There is no specific analysis of the benefit of abemaciclib according to different levels of ER expression. Subgroup analysis showed that abemaciclib was also effective in PR-negative and grade III tumors, which might represent impaired ER-pathway as ERlo cases. More data on this subject is necessary before definitive recommendations regarding the use of adjuvant CDK4/6i in the ERlo population.

Therefore, without prospective evidence and considering the conflicting data from retrospective series, administration of adjuvant ET should be considered on a case-by-case basis in these situations. At the 17th St. Gallen International Breast Cancer Consensus in 2021 (7), the panel was equally divided on the optimal ER threshold for endocrine therapy initiation.

In the advanced disease setting, data about the efficacy of ET in ERlo tumors are virtually non-existent. The combination of CDk4/6i plus ET, the current standard first- and second-line regimens in ERpos metastatic breast cancer (MBC), showed to retain efficacy regardless of ER and PR levels. Still, it should be considered that the limited analysis supporting this statement come from the classification of ER expression on quartiles, and no specific analysis on the use of CDK4/6i in ERlo patients has been reported. At the same time, evidence comparing ET and CT in ERpos MBC is scarce, and no specific subgroup analysis in ERlo patients has been presented.

According to the last European Society of Medical Oncology (ESMO) guidelines, patients with ERlo MBC should not receive ET monotherapy. Instead, they could be considered patients with TNBC for clinical trials, while the administration of CDK4/6i plus ET should remain an option (12). Nonetheless, the same guidelines recommend considering the use of ET whenever ER is positive in at least one biopsy, even in case of discordance between ER expression in primary and metastatic samples. Biological variables of both primary and metastatic samples, along with tumor- and patient-related clinical features, response to previous therapies, the burden of disease, and symptoms, are all elements of significant importance when defining the optimal therapeutic strategy for each patient with ERlo MBC.

Recently, immunotherapy has been approved for treating TNBC in combination with NACT in early-stage and LABC as well as for first-line treatment of MBC. Despite the traditional view of ERpos cancers as non-immunogenic, the enrichment of sTIL and the associated reduced mortality within ERlo cases may suggest an active role of antitumor immunity and the potential of immunotherapy. Even though the phase III trials only included ER-negative disease, it can be hypothesized that ERlo tumors may derive benefits from immune checkpoint blockade similarly to ERneg tumors considering these tumors’ genomic and phenotypic characteristics. This concept has important implications for current clinical practice and the development of clinical trials. In our view, ERlo tumors should be considered for clinical trials evaluating treatment strategies for TNBC. Interestingly, the recently presented neoPACT trial evaluating treatment with pembrolizumab and carboplatin plus docetaxel in TNBC allowed the inclusion of ERlo patients, who represented 15% of the study population (14).

At the same time, the use of PARP inhibitors (PARPi) in patients with germline BRCA1/2 mutation (gBRCAm) is the standard of care in the metastatic and adjuvant settings of high-risk patients with HER2-negative breast cancer (44). The use of adjuvant Olaparib in gBRCAm patients was evaluated in the OlympiA trial, and the inclusion criteria were different for the ER+ positive population and TNBC (45). For ERlo tumors, it is tempting to consider using the eligibility criteria for TNBC rather than those for ERpos disease, thereby allowing more patients with some ER expression to benefit from the PARPi. Since ERlo tumors are comparable to TNBC in terms of BRCA1/2 mutation prevalence (27), these subgroups of tumors probably share features of genome instability and homologous recombination repair deficiency. A trial of neoadjuvant Olaparib and durvalumab (NCT035594396) is ongoing.

Various ongoing trials are also following this concept, and patients with ERlo breast cancer are being included in trials evaluating innovative therapies such as PARP inhibitors in combination with durvalumab (NCT 0359594369), the MEK inhibitor selumetinib (NCT01313039), and EGFR inhibitors. Interestingly, a phase II clinical trial evaluating the role of the anti-EGFR afatinib in combination with letrozole was designed to specifically include patients with ERlo HER2-negative MBC (NCT 02115048).

## Future perspectives

Identifying and treating ERlo BC is challenging for pathologists and oncologists. Analysis of molecular signatures and standardization of therapeutic strategies are essential to understand the biology of ER-low-positive tumors and to enable optimal therapy in the pursuit of personalized medicine. Limited clinical data (mainly derived from retrospective analysis) are available in this subgroup of patients, and it is unlikely that prospective randomized trials will ever be conducted in this population to solve this dilemma. Clinicians should be aware of and able to discuss with patients the limited data on ERlo cases and the interpretability of test results, as stated by the recently updated ASCO/CAP guidelines. Each case should be discussed in a multidisciplinary tumor board, considering both biological and clinical variables.

Integrating innovative molecular biology tests into current clinical practice presents challenges and opportunities. At the same time, validating novel assays and methodologies that are potentially more efficient compared to traditional IHC testing remains an unmet need. In this regard, digital pathology, artificial intelligence, deep learning, and imaging analysis algorithms are innovations that are being progressively incorporated into clinical practice and could improve our ability to classify patient tumors better and optimize our personalized approach to the disease (19). Naik et al. recently demonstrated that machine learning could determine molecular marker status, such as ER expression, directly from cellular morphology (46). The researchers developed a multiple instances learning-based deep neural network that determines ER status from hematoxylin and eosin (H&E)-stained whole slide images. The developed algorithm—trained strictly with WSI-level annotations—is accurate on a varied, multi-country dataset of 3,474 patients and achieved an area under the curve (AUC) of 0.92 for sensitivity and specificity. The authors concluded that this approach could potentially increase clinicians’ capabilities in cancer prognosis and theragnosis by harnessing biological signals imperceptible to the human eye. At the same time, Shamai et al. evaluated tissue microarray H&E stained images from 5356 patients with breast cancer and demonstrated that molecular biomarker expression was significantly associated with tissue characteristics (47). A deep learning model could predict ER expression solely from H&E-stained images with non-inferior accuracy to standard IHC.

Therefore, it is clear that morphological-based molecular profiling has the potential to be implemented into clinical practice as a general approach for mass-scale molecular profiling based on digitized standard H&E-stained images, allowing accurate, fast, and relatively inexpensive methods for the simultaneous profiling of diverse biomarkers in cancer tissues. Integrating ER IHC digital imaging analysis into a busy clinic digital workflow is feasible and may save time and labor for pathologists (48). It is important to emphasize that these methods have not yet been validated for incorporation into routine clinical practice and are not recommended by international guidelines. Artificial neural networks are powerful tools for data analysis and are particularly useful for modeling relationships between variables for the best prediction of an outcome. While these models can be used to answer many important research questions, their utility remains critically limited due to various issues regarding interpretability, technical challenges, and model validation (49).

From a clinical point of view, available data suggest that this subgroup of patients is less dependent on ER pathway signaling and that chemotherapy should be a primary therapeutic strategy. Whether to offer endocrine therapy as part of the overall strategy under the possibility of some remaining endocrine sensitivity should remain an individual discussion. Importantly, we should consider this group as being composed of a heterogeneous number of tumors yet to be fully characterized from the molecular point of view.

## Conclusion

In summary, ERlo tumors represent a relatively small subgroup of breast cancer patients, with an estimated prevalence of 2-5%. These tumors are similar to ERneg disease in their molecular landscape, clinicopathological characteristics, prognosis, and response to therapy. Nevertheless, a proportion may retain some degree of ER signaling dependency, and the possibility of responding to some degree to endocrine therapy cannot be completely ruled out. Molecular tools will probably be required to dissect this complexity and heterogeneity.

Ultimately, attention to estrogen expression levels is essential in clinical practice. Given the potential impact on treatment selection and outcomes, we need caution while considering the optimal management of these patients. Without standard guidelines and recommendations, future research is required in order to develop novel prognostic and predictive biomarkers, optimize current endocrine therapy, explore the potential benefits of chemotherapy, and use immunotherapies and other innovative agents for these patients. Analyzing ongoing trials according to receptor expression levels will help generate further information on the best management strategies for these patients. Simultaneously, further large-scale clinical trials are needed to explore the essential characteristics and sensitivity of ERlo breast cancer to systemic therapies.

## Author contributions

All authors listed have made a substantial, direct, and intellectual contribution to the work and approved it for publication. TR conceived and designed the structure of the manuscript. TR, FC, CAAR, ACG, VPG reviewed the literature and wrote the manuscript. TR and CHB performed guidance and reviews during the writing of the manuscript.

## Conflict of interest

The authors declare the following potential conflicts of interest, although none are directly related to the writing and publication of the present manuscript. TR- Research funding: AstraZeneca, Libbs Speaker honoraria: AstraZeneca, Pfizer, Novartis, MSD, Daichi-Sankyo, Libbs, Lily Advisory board: AstraZeneca, Daichii-Sankyo, Novartis, MSD. CR- Speaker honoraria: AstraZeneca, BMS, Daiichi-Sankyo, Lilly, MSD, Novartis, Pfizer, Roche, United Medical Advisory board: BMS, Roche. AG- Speaker honoraria: Roche, Novartis, Eisai, Eli-Lilly, Pfizer; Daichii Sankyo, Astrazeneca Advisory board: Roche, Eli-Lilly, United Medical, Pfizer. CB- Stock and Other Ownership Interests: Biomarker, MedSIR, Tummi Speaker Honoraria: Novartis, Roche/Genentech, Pfizer, GlaxoSmithKline, Sanofi, Boehringer Ingelheim, Eisai Consulting or Advisory Role: Boehringer Ingelheim, Roche/Genentech, Novartis, GlaxoSmithKline, Eisai, Pfizer, AstraZeneca, Libbs, MSD Oncology, United Medical Research Funding: Pfizer, Novartis, Amgen, AstraZeneca, Boehringer Ingelheim, GlaxoSmithKline, Roche/Genentech, Lilly, Sanofi, Taiho Pharmaceutical, Mylan, Merrimack, Merck, AbbVie, Astellas Pharma, Biomarin, Bristol-Myers Squibb, Daiichi Sankyo, Abraxis BioScience, AB Science, Asana Biosciences, Medivation, Exelixis, ImClone Systems, LEO Pharma, Millennium, Janssen, Atlantis Clinica, INC Research, Halozyme, Covance, Celgene, inVentiv HealthT Travel, Accommodations, Expenses: Roche/Genentech, Novartis, Pfizer, BMS Brazil, AstraZeneca, MSD Oncology.

The remaining authors declare that the research was conducted in the absence of any commercial or financial relationships that could be construed as a potential conflict of interest.

## Publisher’s note

All claims expressed in this article are solely those of the authors and do not necessarily represent those of their affiliated organizations, or those of the publisher, the editors and the reviewers. Any product that may be evaluated in this article, or claim that may be made by its manufacturer, is not guaranteed or endorsed by the publisher.
